# Blood leukocytes methylation levels analysis indicate methylated plasma test is a promising tool for colorectal cancer early detection

**DOI:** 10.7150/jca.57114

**Published:** 2021-04-30

**Authors:** Zhiliang Chen, Guodong Zhao, Kai Wang, Xiaomei Wang, Yong Ma, Shangmin Xiong, Minxue Zheng, Sujuan Fei

**Affiliations:** 1 Department of Gastroenterology, Affiliated Hospital of Xuzhou Medical University, Xuzhou Jiangsu 221002, China; 2 Institute of Digestive Diseases, Xuzhou Medical University, Xuzhou Jiangsu 221002, China; 3 Zhejiang University Kunshan Biotechnology Laboratory, Zhejiang University Kunshan Innovation Institute, Kunshan Jiangsu 215300, China; 4 State Key Laboratory of Bioelectronics, School of Biological Science and Medical Engineering, Southeast University, Nanjing 210009, China; 5 Suzhou Institute of Biomedical Engineering and Technology, Chinese Academy of Sciences, Suzhou Jiangsu 215163, China; 6 Suzhou VersaBio Technologies Co. Ltd., Kunshan Jiangsu 215300, China

**Keywords:** Colorectal cancer, plasma, leukocyte, m*SEPT9*, * mSDC2*, early detection

## Abstract

**Background**: A number of plasma methylated DNA biomarkers related to colorectal cancer (CRC) have been identified. However, the effect of methylation level in leukocytes on plasma-based methylation test was rarely reported.

**Methods**: Blood samples from 213 individuals including 91 CRC patients were collected and separated into 3.5 mL of plasma and paired leukocyte fractions. DNA were extracted from plasma and leukocytes and bisulfite converted, followed by ColoDefense test that detects methylated *SEPT9* (m*SEPT9*) and methylated *SDC2* (m*SDC2*) simultaneously in a single qPCR reaction.

**Results**: Both m*SEPT9* and m*SDC2* levels in leukocytes exhibited no significant difference among CRC, benign tumors and healthy controls. However, m*SEPT9* and m*SDC2* levels in plasma were significantly higher in CRC group than those in other groups. The sensitivities of m*SEPT9* and m*SDC2* alone for detecting CRC with plasma samples were 75.8% and 60.4% with specificities of 94.7% and 86.8%, respectively. These two markers in combination exhibited an improved sensitivity of 85.7% for CRC detection with a specificity of 86.8%, mostly attributable to increased sensitivity of 81.8% for detecting stage 0-II CRC. AUC values for m*SEPT9* and m*SDC2* alone were 0.864 (95% CI: 0.798 - 0.929) and 0.796 (95% CI: 0.719 - 0.874), respectively, but improved to 0.972 (95% CI: 0.949 - 0.996) when combined for ColoDefense test.

**Conclusions**: The leukocytes gDNA will not affect the performance of plasma ColoDefense test, and plasma ColoDefense test exhibited high sensitivity and specificity in a validation set, demonstrating its potential as a non-invasive and cost-effective method for CRC early detection.

## 1. Introduction

Liquid biopsy, primarily blood test, provides potential non-invasive screening approaches to assess many diseases including cancers. Peripheral blood is a rich library of cells and genetic materials, capable of providing real-time information originating from primary or metastatic tumor sites. The major analytes for identifying and quantifying genetic and epigenetic information in peripheral blood liquid biopsy include circulating cell-free DNA (cfDNA), circulating tumor cells (CTC), messenger RNA (mRNA), microRNA (miRNA) and leukocytes, etc. Each type of analyte has its own potentials and drawbacks in addressing specific needs. For example, CTCs provide intact cancer-specific information at genome, RNA and protein levels, making them a powerful tool to detect cancers in advanced stages and a reliable prognostic biomarker 1. However, its rareness in early stage cancer and high cost in enrichment, detection and characterization made it a less prevailing screening tool in the near future.

Among these analytes, cfDNA biomarker testing is one of the most functional and promising technologies in screening, diagnosis and prognosis of cancers and other diseases. For healthy individuals or patients in the early stage of tumorigenesis, the amount of cfDNA is estimated to be approximately 3-9 ng/mL of plasma, while there could be a 10-fold increase in the concentration of plasma cfDNA for patients with advanced cancer 2. Main origins of cfDNA include endogenous DNA from cellular structures in circulation or nervous system that contain or release DNA during normal or abnormal activities into circulation, and exogenous DNA entering the human body via infection, ingestion, inhalation, transplant, transfusion, or as fetal DNA fragments, etc. The interaction of these activities may further stimulate the release of cfDNA in circulation 3. Of these sources, blood cells, especially leukocytes, contribute a large amount of cfDNA in healthy subjects 4.

However, recent studies have also shown that considerable proportion of somatic mutations identified in leukocyte genomic DNA (gDNA) in individuals with or without cancer were also present in cfDNA, raising the possibility of false positive in detection of cancer-derived mutations 5 6. In addition, DNA and RNA sequence analysis indicated that somatic mutations exist across normal tissues in varying degrees, and genetic clones containing such mutations may not have the potential to develop into cancers 7, the widespread presence of mutations in normal tissues and cancerous lesions makes them a less valuable biomarker candidate in early cancer detection. Aberrant methylation of specific genes has been shown to be associated with many cancer types including CRC 8. A number of abnormally methylated genes related to CRC have been identified, including methylated *SEPT9*, *SDC2*, *CLIP4* and *SFRP2*, some of which have been commercialized for CRC screening 9-14. *SEPT9* is the coding gene for *septin-9*, a protein involved in cytokinesis, and its hypermethylation in CRC makes it a useful biomarker. *SDC2* encodes *syndecan-2*, a glycoprotein involved in cell binding, signaling and cytoskeletal organization. Previous studies of methylated *SDC2* (m*SDC2*) showed it to be a promising biomarker for both stool- and plasma-based CRC screening test 14-16. Recently, a new plasma-based methylation test (ColoDefense test) for CRC early detection was reported, which contained both methylated *SEPT9* (m*SEPT9*) and m*SDC2* simultaneously in a single qPCR reaction with high sensitivity and specificity 14. However, whether the plasma methylated DNA derived from leukocyte gDNA will cause the false positive in CRC detection or not was rarely reported. Here, we report the results of a study of the methylation levels of *SEPT9* and *SDC2* in plasma and leukocytes of patients with CRC or precancerous lesions and health subjects. Our primary goal was to evaluate the effect of methylation levels for* SEPT9* and *SDC2* in leukocytes on the performance of ColoDefense test, and our secondary aim was to further validate the plasma test for CRC early detection in a validation set.

## 2. Materials and Methods

### 2.1. Sample collection

**Figure 1:**
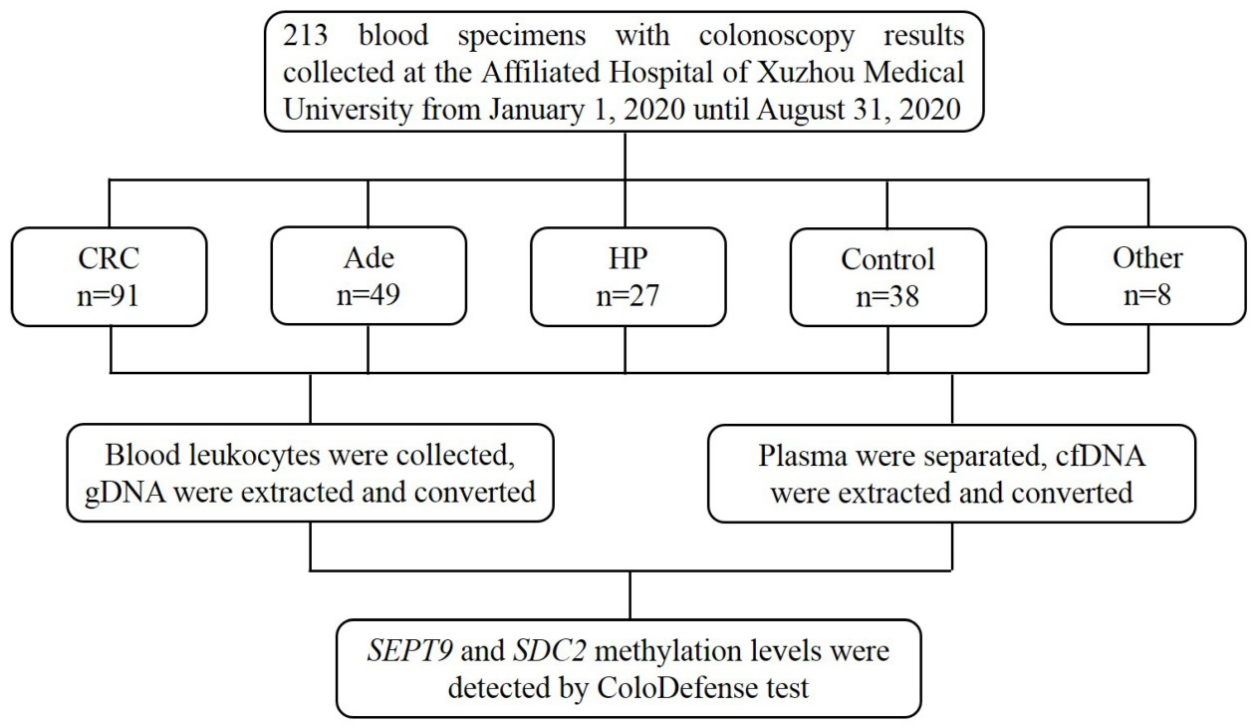
The flow chat of this study.

Blood specimens were collected at the Affiliated Hospital of Xuzhou Medical University from January 1, 2020 to August 31, 2020. The inclusion criteria consisted of the following: aged 18 or older, no history of CRC, no pregnant woman, having colonoscopy results, and participants with abnormal colonoscopy results should have pathological diagnosis results. The exclusion criteria were as follows: missing or incomplete sample information, insufficient blood volume, repeated sampling, severe hemolysis, and insufficient DNA indicated by low *ACTB* levels (see data analysis). Finally, 213 blood samples were collected, including 91 from CRC patients, 49 from adenomatous polyp patients (Ade), 27 from hyperplastic polyp patients (HP), 38 from control subjects with normal colonoscopy results and 8 from patients with other gastrointestinal tumors (Figure 1). Ten milliliter blood was drawn from each subject and stored at 4℃ for no more than 24 hours. The blood samples were centrifuged at 1,350 g for 12 min to separate plasma and leukocytes. The 3.5mL plasma fractions and leukocyte fractions were then frozen at -80℃ until use. This study was approved by the Institutional Review Board of the Affiliated Hospital of Xuzhou Medical University (Ethics Committee reference number: XYFY2020-KL123-01), and informed consent was obtained from all participating patients and control subjects.

### 2.2. DNA extraction and bisulfite conversion

Leukocyte genomic DNA was isolated with VersaPrep DNA extraction kit (Suzhou VersaBio Technologies Co. Ltd., Kunshan, Jiangsu, China). Specifically, 250μL lysis buffer and 20μL proteinase K solution were added to each leukocyte specimen and followed by incubation at room temperature for 10 min. Afterwards, 180μL ethanol was added into each sample and the mixture was loaded onto a spin column. After two washing steps, the purified genomic DNA was eluted with 100μL elution buffer. Plasma circulating free DNA (cfDNA) was extracted using a cfDNA extraction kit (Suzhou VersaBio Technologies Co. Ltd.) according to previously published procedure 14. Subsequently, 100 μL purified cfDNA and leukocyte gDNAwere used for bisulfite conversion and purification by a fast bisulfite conversion kit (Suzhou VersaBio Technologies Co. Ltd.) 14.

### 2.3. Quantitative real-time PCR

Converted and purified DNA from leukocytes and plasma were examined by ColoDefense test (Suzhou VersaBio Technologies Co. Ltd.), a multiplex qPCR assay detecting m*SEPT9*, m*SDC2* and *ACTB* simultaneously in one qPCR reaction 14. Each plasma cfDNA sample was tested in three qPCR replicates, and a single qPCR reaction was performed for each leukocyte gDNA sample. The qPCR was performed on LC480-II thermal cycler (Roche Diagnostics) with the following cycling conditions: activation at 95°C for 30 minutes, 50 cycles of 95°C for 10 seconds, 58°C for 30 seconds, 72°C for 10 seconds, and final cooling to 40°C for 30 seconds 14.

### 2.4. Data analysis

For a plasma sample, the result was considered 'invalid' if *ACTB* Ct was greater than 35.0, and m*SEPT9* and m*SDC2* were considered 'detected' if their Ct values were less than 45.0 and 50.0, respectively. m*SEPT9* and m*SDC2* were scored positive respectively by 1/3 and 2/3 rules. A plasma sample would be scored positive for ColoDefense test if either m*SEPT9* or m*SDC2* was positive 14. ∆Ct was used to determine the methylation levels of *SEPT9* and* SDC2* genes in leukocytes. ∆Ct was defined as the difference between the Ct values of the target (m*SEPT9* or m*SDC2*) and the internal control gene (*ACTB*) 12. Mean Ct values of m*SEPT9* and m*SDC2* were used to determine the methylation levels in plasma samples. For qPCR reactions without detectable amplification signals, their Ct values were set at 50, the maximal number of PCR cycles. Mean Ct values of m*SEPT9* and m*SDC2* for CRC patients and control subjects were also used to plot ROC curves. Data were subjected to statistical analysis with IBM SPSS software for Windows Version 22.0. *t*-test was used for comparison of two groups, and Pearson chi-square test was used for comparison among more than two groups. The differences of methylation levels were analyzed by Mann-Whitney U test.

## 3. Results

The characteristics of 213 subjects enrolled in this study are shown in Table 1. The mean age of Ade and HP patients was 54.6 and 52.5 with 79.6% and 59.3% males, respectively. Ninety-one CRC patients were enrolled including 4 stage 0, 9 stage I, 31 stage II, 29 stage III, 4 stage IV and 14 of unknown stage. Other tumor groups included 5 rectal neuroendocrine tumor patients, two rectal melanoma patients and one gastric stromal tumor patients.

**Figure 2:**
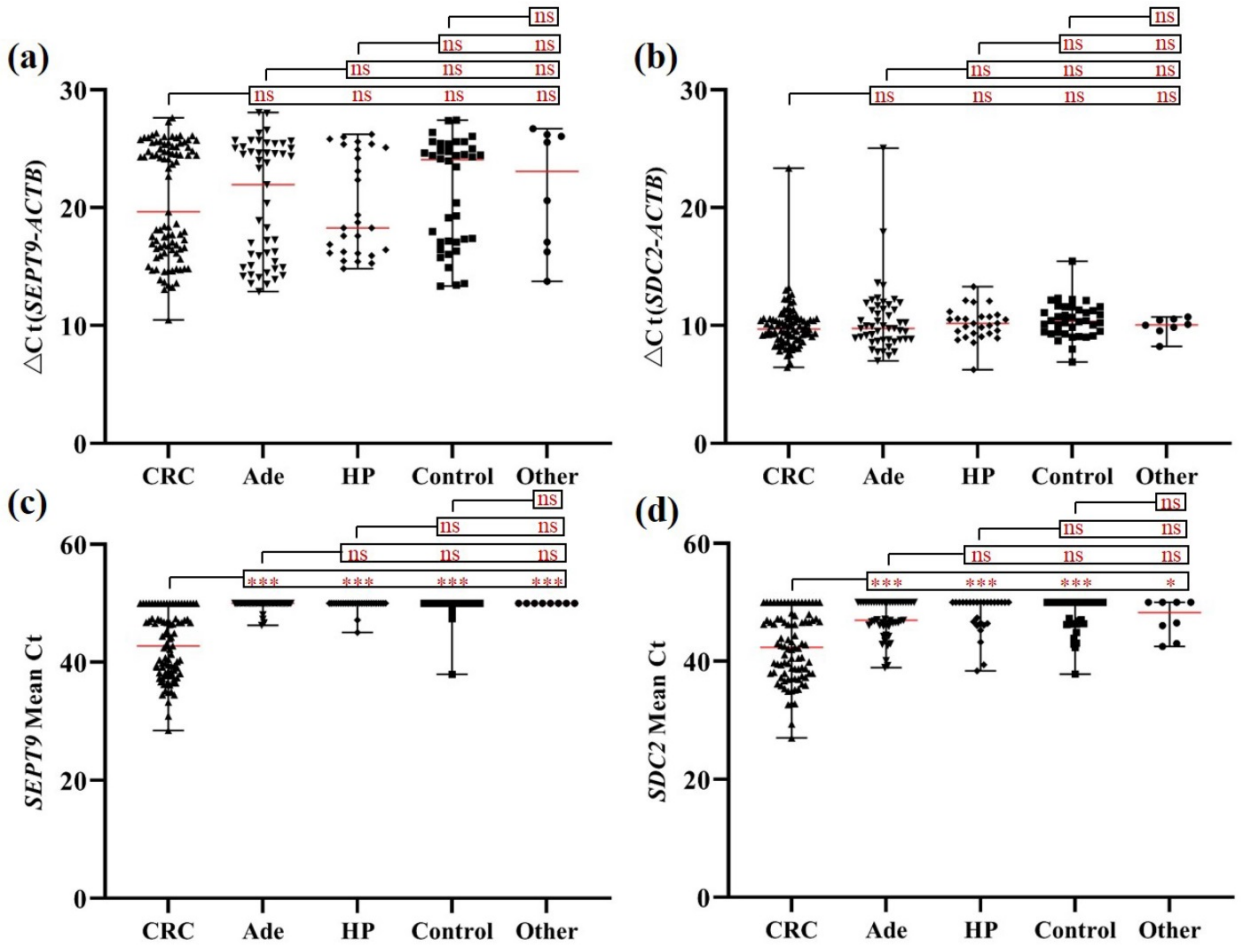
m*SEPT9* (a, c) and m*SDC2* (b, d) levels in blood leukocytes (a, b) and plasma (c, d) from CRC, Ade, HP, and other tumor patients as well as control subjects. ns, not significant. *, *p* < 0.05. **, *p* < 0.01. ***, *p* < 0.001. Red lines represent the median methylation levels of* SEPT9* and *SDC2.*

As shown in Figure 2, m*SEPT9* (Figure 2a) and m*SDC2* (Figure 2b) levels in blood leukocytes from CRC, Ade, HP, other tumor and control groups exhibited no significant difference. In contrast, m*SEPT9* and m*SDC2* levels in CRC plasma were significantly higher when compared to those of Ade, HP, other tumor and control groups, which showed no significant difference among themselves (Figures 2c and 2d).

**Figure 3:**
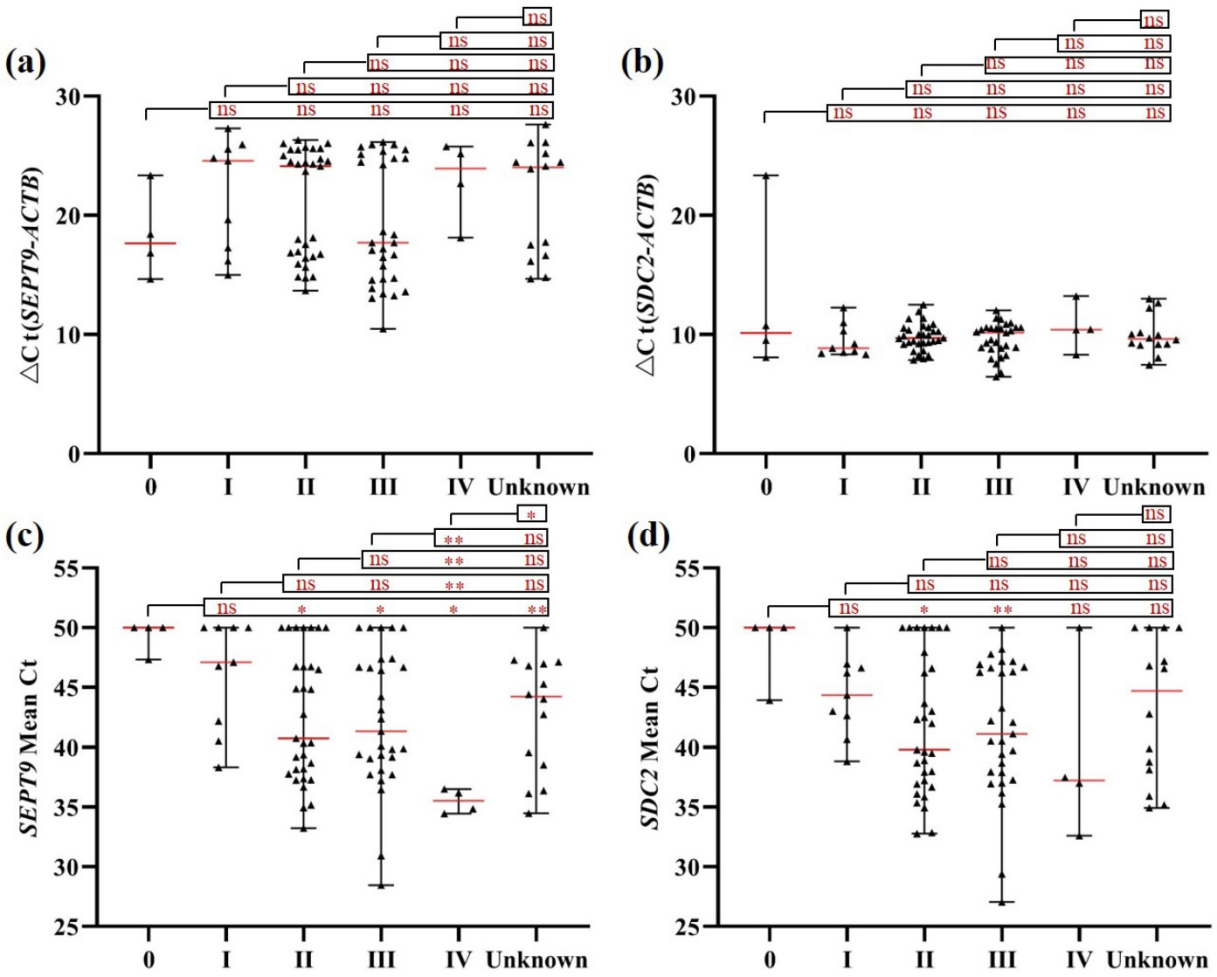
m*SEPT9* (a, c) and m*SDC2* (b, d) levels in blood leukocytes (a, b) and plasma (c, d) from CRC patients of different stages. ns, not significant. *, *p* < 0.05. **, *p* < 0.01. ***, *p* < 0.001. Red lines represent the median methylation levels of* SEPT9* and *SDC2.*

Moreover, blood leukocyte m*SEPT9* and m*SDC2* levels both showed no significant difference among CRC patients of different stages (Figures 3a, 3b). For plasma samples, both *SEPT9* and *SDC2* showed higher methylation levels in stages II-III CRC patients when compared to stage 0 patients (Figures 3c and 3d). Furthermore, stage IV patients also displayed higher m*SEPT9* level than patients of other stages (Figure 3c), but such a trend was not observed for m*SDC2* levels (Figure 3d).

**Figure 4:**
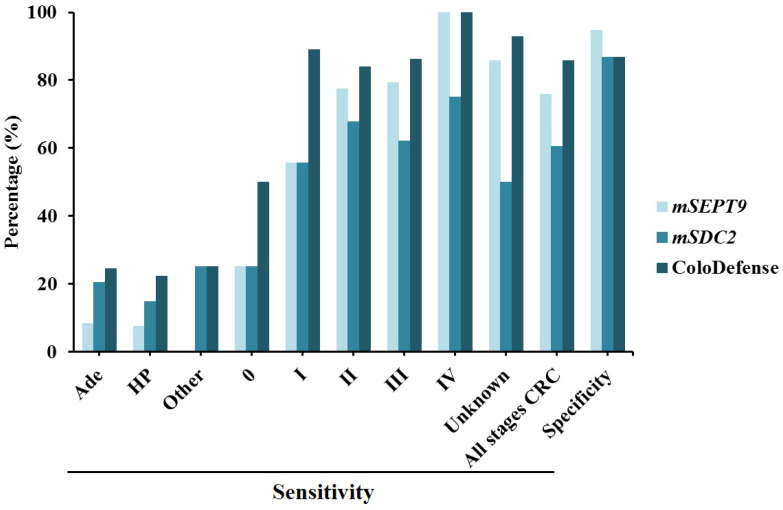
The sensitivities and specificities of plasma-based assay of m*SEPT9* alone, m*SDC2* alone and ColoDefense test for the detection of Ade, HP, other tumors and different stages of CRC.

This study also displayed a validation results for plasma ColoDefense test in detecting CRC. The sensitivities of m*SEPT9* alone and m*SDC2* alone for CRC detection were 75.8% and 60.4% with specificities of 94.7% and 86.8%, respectively. When m*SEPT9* and m*SDC2* were combined as in ColoDefense test, the sensitivity was improved to 85.7% with a specificity of 86.8% (Figure 4). The sensitivity improvement was mainly due to higher positive detection rates of early stage (stage 0-II) CRCs. The sensitivities of m*SEPT9* alone and m*SDC2* alone for stage 0-II CRC detection were 68.2% and 61.3%, but ColoDefense test showed a 81.8% sensitivity, representing 13.6% and 20.5% increase over those of each biomarker alone. The sensitivities of ColoDefense test for detecting Ade, HP and other gastrointestinal tumors were 24.5%, 22.2% and 25.0%, respectively, which were significantly lower than that for CRC detection (Figure 4).

**Figure 5:**
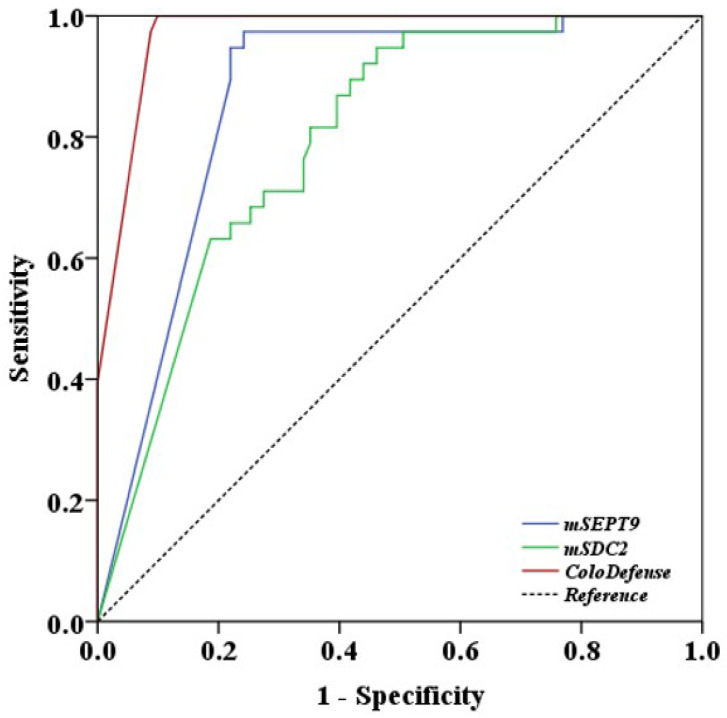
ROC curves for plasma m*SEPT9* test alone, m*SDC2* test alone and the combined ColoDefense test for detecting CRC.

Finally, receiver operating characteristic (ROC) curve analysis was performed with plasma test results. Area under the curve (AUC) value for ColoDefense test in detecting CRC was 0.972 (95% CI: 0.949 - 0.996), whereas AUC values for m*SEPT9* alone and m*SDC2* alone were 0.864 (95% CI: 0.798 - 0.929) and 0.796 (95% CI: 0.719 - 0.874), respectively, 0.108 and 0.176 lower when compared to the combined ColoDefense test (Figure 5). The sensitivities of m*SEPT9* alone, m*SDC2* alone and ColoDefense test for detecting CRC of different characteristics exhibited no significant difference among different age groups, genders, T or Nstages, tumor sizes, tumor sites and degrees of differentiation. The only exception was that the sensitivities of m*SEPT9* alone showed significant difference for different T stages (Table 2), which was likely due to the fact that T stage represents the size and location of primary tumors so that early T stage CRCs usually have smaller sizes and thus release less DNA into the circulation, resulting in lower sensitivity. However, adding m*SDC2* detection to the test could compensate for the low performance of single m*SEPT9* biomarker in this aspect.

## 4. Discussion

Ranked as the third most common and the second most deadly cancer type, CRC has attracted growing attention in the past decades 17. CRC mortality could be largely mitigated if appropriate diagnosis, especially of precancerous lesion, and surveillance were performed in time 18. Substantially reduced morbidity and mortality rates in the US during the past decade were largely attributed to the high prevalence of CRC screening among adults 50 years and older, as high as 61% of who have undergone a colonoscopy 19. However, the invasiveness of colonoscopy, its tedious procedure and relatively high demand for medical resources have resulted in low compliance rate especially among low-to-average risk population in most countries. In this study, a plasma-based multi-target DNA methylation test was shown to be a potential cost-effective solution to this dilemma.

Multiple methylation biomarkers for precancerous lesion and CRC screening have been evaluated independently or in combination in plasma samples. The first blood based m*SEPT9* assay approved by FDA, Epi proColon 2.0 assay, exhibited limited sensitivities of 22% and 68.2% for precancerous lesions and CRCs, respectively, with a specificity of 78.2% using 1/3 scoring algorithm 20. Other retrospective case control studies based on plasma m*SEPT9* showed a sensitivity of ~70% and a specificity of ~90%, while a prospective study in 7941 asymptomic individuals showed a sensitivity of 48.2% and a specificity of 91.5% 21, 22. In our study, the sensitivity of m*SEPT9* alone for CRC detection was 75.8% with a specificity of 94.7%, in accordance with previous retrospective studies. m*SDC2* was also suggested as a candidate blood-based biomarker in a panel study, and its performance was evaluated alone or more often when combined with other methylated markers, demonstrating its potential contribution in combination tests 23, 24, 9. In the present validation study of plasma ColoDefense test, combining m*SDC2* with m*SEPT9* resulted in an improvement of 9.9% in sensitivity with a specificity of 86.8%, in good agreement with our previous study that showed 88.9% sensitivity (95% CI: 81.4%‐93.7%) and 92.8% specificity (95% CI: 87.4%‐96.0%) in a training set 14. Therefore, consistent performance of our multiplex DNA methylation test with plasma samples in independent cohorts further validated its potential as an alternative approach for CRC screening.

In general, plasma-based tests for screening are preferred over whole blood mainly because inclusion of blood cells would introduce excessive amount of DNA/RNA as background. Meanwhile, changes in epigenetic characteristics of peripheral blood leukocyte gDNA could directly reflect the status of immune responses triggered by tumor genesis, development, progression and remission. Previous studies of the methylation level of leukocyte gDNA have identified markers that were in concordance with several cancer types or precancerous lesions, including colorectal adenomas, CRC, gastric cancer and breast cancer 25-27. For example, Zhang* et al.* showed that the methylation levels of *IGFII* and *N33* and the frequency of m*IGFII* were significantly higher in gastric cancer cases than those of a high-risk population, suggesting their association with gastric carcinogenesis 28. Another study showed that a combination of three methylated markers in *KIAA1549L* and *BCL2* genes yielded a c-statistics value of 0.69 in a CRC screening test, less impressive in comparison with other established markers 29. In this study, no significant differences in the methylation levels of *SEPT9* and *SDC2* in leukocytes were observed among CRC, Ade, HP and control groups, while either m*SEPT9* or m*SDC2* level alone in plasma were able to distinguish CRC cases from patients with non-cancerous lesions or healthy subjects. These observations indicated that the ratio of certain methylated genes in plasma differed from that in leukocytes, the main proportion of m*SEPT9* and m*SDC2* in plasma was originally from cancer tissues rather than leukocytes, and the leukocytes gDNA will not affect the performance of plasma ColoDefense test. Meanwhile, the overwhelming amount of gDNA in leukocytes suggests analyzing methylation levels of candidate genes in both leukocytes and plasma during marker selection to avoid false positive caused by hypermethylation of certain markers only in leukocytes.

Compared with m*SEPT9* level in leukocytes, m*SDC2* level was orders of magnitude higher with much lower ΔCt value, suggesting an increased amount of leukocyte m*SDC2* released into plasma that could account for the 7.9% lower specificity. This further emphasizes that care should be taken to avoid additional blood cell damages when separating plasma and leukocytes. In addition, such a scenario could also account for the optimized 2/3 scoring algorithm for m*SDC2* in comparison to 1/3 algorithm for m*SEPT9* as 2/3 algorithm should reduce the interference from accidental rupture of the leukocytes more than 1/3 algorithm.

## 5. Conclusion

In this study, we evaluated the methylation levels of *SEPT9* and *SDC2* in peripheral blood leukocytes and plasma from patients with CRC and precancerous lesions as well as healthy subjects. Our results showed that the methylation levels of both markers in plasma could differentiate CRC from benign tumors and healthy controls, while their levels in leukocytes could not. Furthermore, the test results indicated that the overall sensitivity for CRC, especially early stage CRC, was greatly improved upon combination of m*SEPT9* and m*SDC2*. In conclusion, plasma ColoDefense test was a promising tool for CRC early detection.

## Figures and Tables

**Table 1:** The characteristics of the subjects enrolled in this study.

Group	Total number	Gender (%)		Age	
		Male	Female	Mean (SD)	Min-Max
Ade	49	39 (79.6)	10 (20.4)	54.6 (11.7)	29-78
HP	27	16 (59.3)	11 (40.7)	52.5 (11.2)	27-77
CRC	91	55 (60.4)	36 (39.6)	62.7 (13.9)	22-89
0	4	0 (0.0)	4 (100.0)	70.3 (10.4)	53-80
I	9	3 (33.3)	6 (66.7)	56.3 (12.3)	30-72
II	31	23 (74.2)	8 (25.8)	60.9 (12.8)	36-85
III	29	18 (62.1)	11 (37.9)	64.5 (12.7)	25-89
IV	4	3 (75.0)	1 (25.0)	62.5 (10.2)	55-80
Unknown	14	9 (64.3)	5 (35.7)	64.7 (18.4)	22-86
Other tumors	8	4 (50.0)	4 (50.0)	49.6 (9.6)	38-68
Rectal neuroendocrine tumor	5	3 (60.0)	2 (40.0)	45.4 (6.8)	38-58
Rectal melanoma	2	1 (50.0)	1 (50.0)	56.5 (11.5)	45-68
Gastric stromal tumor	1	0 (0.0)	1 (100.0)	57 (/)	/
Control	38	20 (52.6)	18 (47.4)	47.2 (14.5)	20-81

SD, standard deviation.

**Table 2:** Sensitivities of plasma m*SEPT9* test alone, m*SDC2* test alone and the combined ColoDefense test for detecting CRC of different genders, age groups, stages, tumor sites, tumor sizes and degrees of differentiation.

	m*SEPT9*		m*SDC2*		ColoDefense	
	Sensitivity (%)	*p*	Sensitivity (%)	*p*	Sensitivity (%)	*p*
**Patients Gender**						
Male (n=55)	74.6	0.725	61.8	0.740	81.8	0.189
Female (n=36)	77.8		58.3		91.7	
**Patients Age**						
<40 (n=7)	57.1	0.075	14.3	0.085	57.1	0.098
40-49 (n=7)	57.1		57.1		85.7	
50-59 (n=20)	80.0		75.0		90.0	
60-69 (n=28	64.3		57.1		78.6	
70-79 (n=21)	90.5		71.4		95.2	
≥80 (n=8)	100.0		50.0		100.0	
**Tumor T stage**						
Tis (n=4)	25.0	0.022	25.0	0.406	50.0	0.254
T1 (n=1)	0.0		100.0		100.0	
T2 (n=13)	61.5		53.9		76.9	
T3 (n=49)	79.6		63.3		85.7	
T4 (n=9)	88.9		77.8		100.0	
N/A (n=15)	86.7		53.3		93.3	
**Tumor N stage**						
N0 (n=45)	68.9	0.472	60.0	0.939	82.2	0.483
N1 (n=23)	78.3		65.2		82.6	
N2 (n=8)	87.5		62.5		100.0	
N/A (n=15)	86.7		53.3		93.3	
**Tumor site**						
Colon (n=29)	79.3	0.513	72.4	0.136	89.7	0.412
Rectum (n=59)	72.9		55.9		83.1	
N/A (n=3)	100.0		33.3		100.0	
**Tumor Size**						
≤4 cm (n=34)	70.6	0.880	58.8	0.845	82.4	0.913
>4 cm (n=36)	72.2		61.1		83.3	
N/A (n=21)	90.5		61.9		95.2	
**Degree of differentiation**						
Low (n=5)	40.0	0.156	80.0	0.587	80.0	0.779
Moderate (n=41)	78.1		56.1		85.4	
High (n=20)	65.0		60.0		75.0	
N/A (n=25)	88.0		64.0		96.0	

N/A, not applicable.

## Data Availability

The datasets analyzed during this study are available from the corresponding author on reasonable request.
